# Natural products and their derivatives as candidate treatments for hair greying: from drug discovery to molecular mechanisms

**DOI:** 10.1186/s13020-026-01340-0

**Published:** 2026-02-03

**Authors:** Chaoying Zhu, Yuan Gao, Haiying Gong, Jiabo Wang

**Affiliations:** 1 School of Pharmacy, Chengdu University of Traditional Chinese Medicine, Chengdu, 611137 China; 2 School of Traditional Chinese Medicine, Capital Medical University, Beijing, 100069 China

**Keywords:** Hair greying, Natural products, Derivatives, Melanocyte stem cells, Melanin synthesis

## Abstract

**Ethnopharmacological relevance:**

Hair greying is a common aspect of the natural ageing process. Although it is generally not considered a medical problem, its high prevalence can substantially impact emotional state due to aesthetic concerns. A growing body of research has demonstrated that natural products and their derivatives derived from plants possess advantages and potential in the treatment of hair greying.

**Aim of the study:**

To review the last research progress in the treatment of hair greying by natural products and their derivatives, focusing on the target and mechanism of action of natural products and their derivatives and providing a reference for future clinical use.

**Materials and methods:**

We searched electronic databases (PubMed, Web of Science, ClinicalTrials.gov, and CNKI) for studies published between January 2005 and June 2025. The research focused on the pathogenesis of hair greying and the use of natural products and their derivatives to prevent and treat it, using the keywords: “hair greying”, “hair pigmentation”, “white hair”, “snow hair”, “melanocyte stem cells” and “melanin”.

**Results:**

Account of natural products (e.g., *Polygoni multiflori* radix) and their derivatives (e.g., Epimedin B), are expected to treat hair greying due to their various qualities to regulate melanocyte stem cells, enhance melanin synthesis, or promote melanosome transport. Compared to oral administration, topical application represents a preferred approach for promoting hair pigmentation.

**Conclusions:**

We discussed and summarized the mechanism of natural products and derivatives in the treatment of hair greying, which provided a reference for future clinical use.

**Graphical Abstract:**

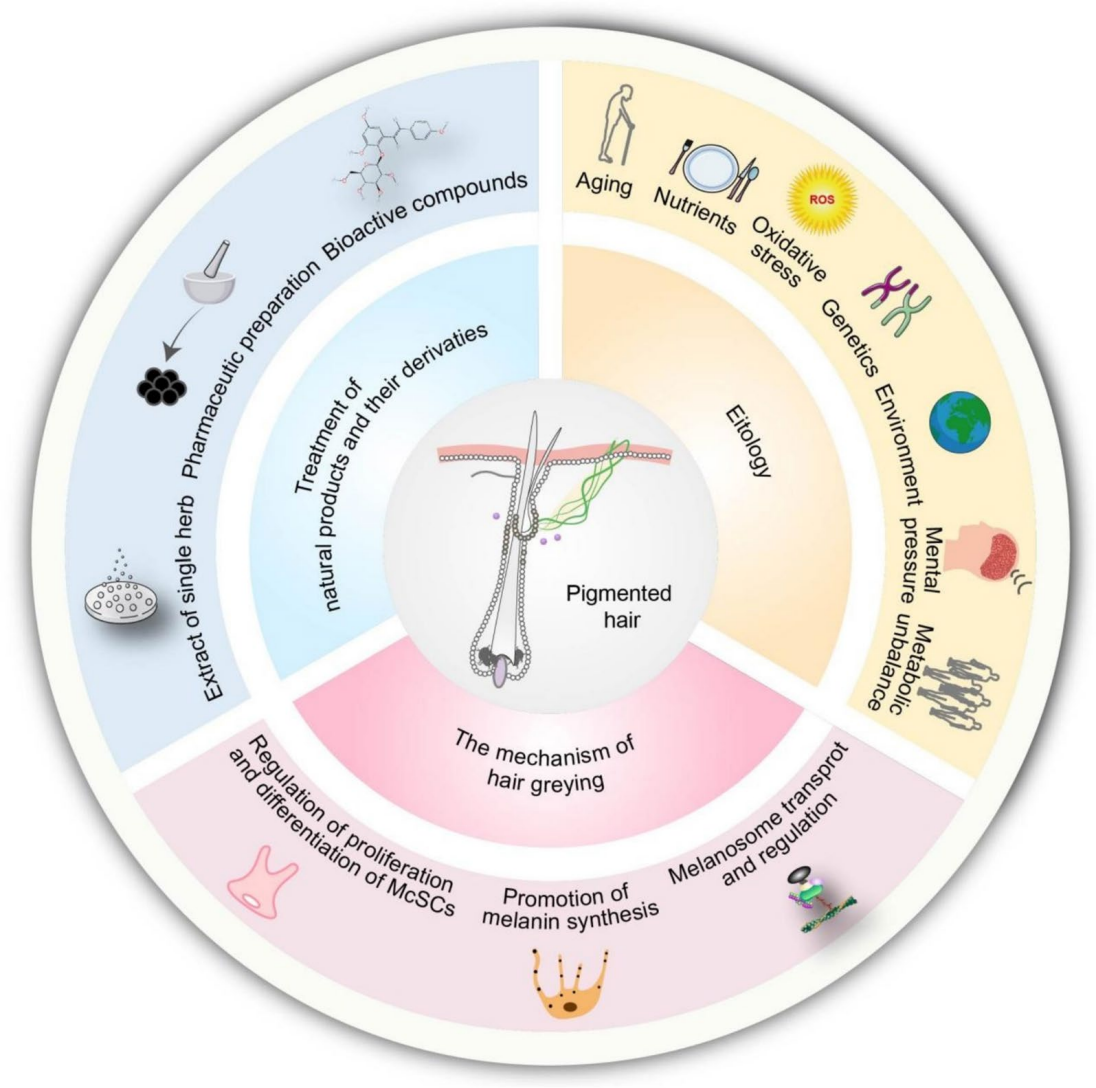

**Supplementary Information:**

The online version contains supplementary material available at 10.1186/s13020-026-01340-0.

## Highlights

1. Hair greying is increasingly common and occurs at a younger age.
2. The pathogenesis of hair greying is related to the abnormal function of melanocyte stem cells, and the abnormalities in melanin synthesis and transportation.
3. Natural products and their derivatives can regulate the function of melanocyte stem cells and promote the normal process of melanin synthesis and transportation, thus helping to colour the hair.
4. Natural products and their derivatives may represent a promising direction for the development of new drugs for treating hair health problems.

**Supplementary Information:**

The online version contains supplementary material available at 10.1186/s13020-026-01340-0.

## Introduction

Most people have a desire for continuous youth, and hair greying is a highly prevalent, visible early sign of human ageing [166]. Typically beginning in the third decade of life, many individuals experience significant or progressive hair loss of pigmentation, known as hair greying [145]. Despite hair greying being typically thought of as a progressive and irreversible age-related process, some studies have documented instances of medication- and mineral-induced hair discolouration [100]. Unpigmented hair can also be sign or symptom of several diseases that are not related to age. Increasing evidence from clinical and animal studies suggest that hair greying is an independent predictor of serious dermatological pathologies and other conditions [200], such as vitiligo [133], cardiovascular diseases[41], oculocutaneous albinism [197], and osteoporosis[167].

In fact, dyeing hair is the first choice. Despite the abundance of hair products on the market, many fail to meet people’s expectations because of water dilution and short contact time with hair [145]. Another example would be popular hair dyes which rely on corrosive chemical processes, resulting in acute or chronic side effects, including dry, split, and dull hair shafts [20], anaphylaxis [76], alopecia [92], urticarial [47], and cancer [112, 270, 276, 277], and they do not address the root cause of hair greying. Therefore, the need to discover and develop more effective strategies for treating hair greying has become urgent.

**Fig. 1:**
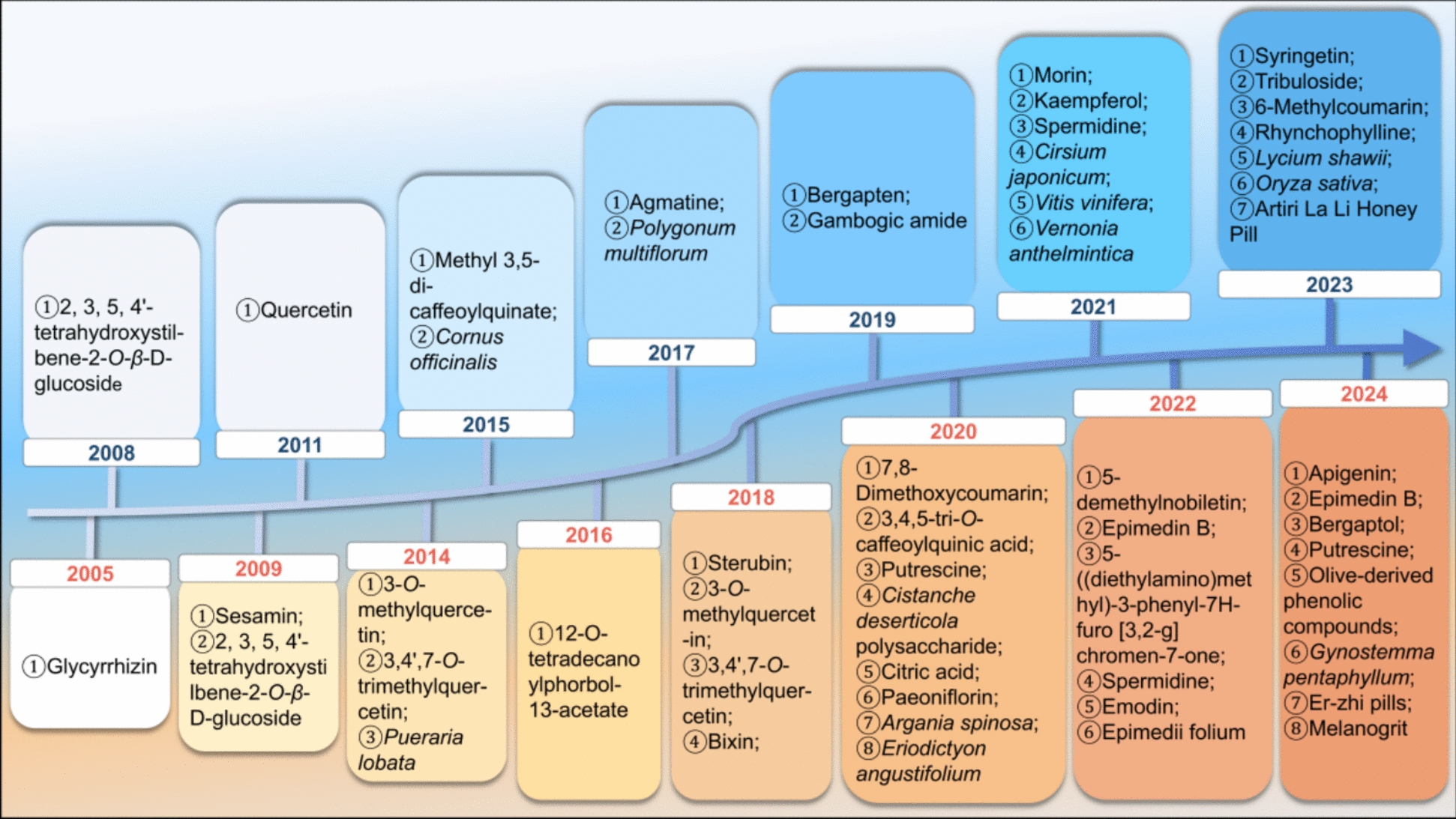
Evolution and milestones of natural products and derivatives management in hair greying. Since 2004, we have conducted a comprehensive search in databases such as PubMed and Web of Science for natural products and their derivatives for the treatment of grey hair and quantified and analysed the number of articles published annually on this topic

Over recent decades, natural products and their derivatives from plants and animals have helped in alleviating the distress caused by hair greying. Owing to their structural diversity, broad range of sources, and biological activity, these compounds hold great prospect for the development of modern drugs targeting hair greying. These natural products and their derivatives have been a key research focus, encompassing a wide range of natural plants, such as polysaccharides [82, 84], alkaloids [34], and flavonoids [72]. They potently promote melanin production by increasing overall melanin content, enhancing tyrosinase (TYR) activity, and up-regulating key molecules involved in melanin synthesis and transport, all without including resistance. However, issues including low bioavailability and instability continue to hinder the clinical application of natural medications in treating hair greying. In this review, we provide insights into the complex molecular and cellular processes involved in the pathogenesis of hair pigmentation. We have reviewed the therapeutic effects of natural products and their derivatives on hair greying and examined the underlying mechanisms of these interventions. We have summarised the functions of natural products and their derivatives in hair pigmentation by modulating the proliferation and differentiation of follicular melanocyte stem cells (McSCs), melanin synthesis, and its transport. Our aim is to provide a comprehensive analysis of the pathological mechanisms underlying unpigmented hair and to elaborate on the role of natural products and their derivatives over the last 20 years in ameliorating hair greying, as well as the potential mechanisms of action. The evolution and milestones of natural products and their derivatives in the management of hair greying are illustrated in Fig. 1. A detailed overview of these aspects could encourage further research and provide new insights into impeding the development of hair greying. The full names of the botanicals referred to in this study have been checked using http://www.worldfloraonline.org on April 15, 2025.

## Pathophysiology of hair greying

The natural coloration of hair is a multi-step process. Melanin, is produced by melanocytes, which are a type of cell differentiated from McSCs. It is responsible for producing pigment for hair under physiological conditions. Generally, in the telogen phase of the hair follicle, McSCs remain quiescent. However, in the early anagen phase, quiescent McSCs are activated through Wnt signalling, which undergo proliferation followed by differentiation producing transit-amplifying (TA) progeny. TA cells form a branch and a portion of TA cells differentiate into mature melanocytes that migrate to the hair bulb of the hair follicle. Here, they synthesise melanin and then melanin is transported to the keratinocytes through the dendrites before colouring the hair in the mid to late anagen phase of the hair cycle. The other portion of undifferentiated TA cells transfer to the bulge and outer root sheath and then back to the bulge during the catagen phase to colour the hair during the next hair follicle cycle [212]. With the depletion or dysfunction of McSCs, the processes of melanin synthesis and transportation are aberrant. Any interference within these processes increases the likelihood of developing grey hair.

**Fig. 2:**
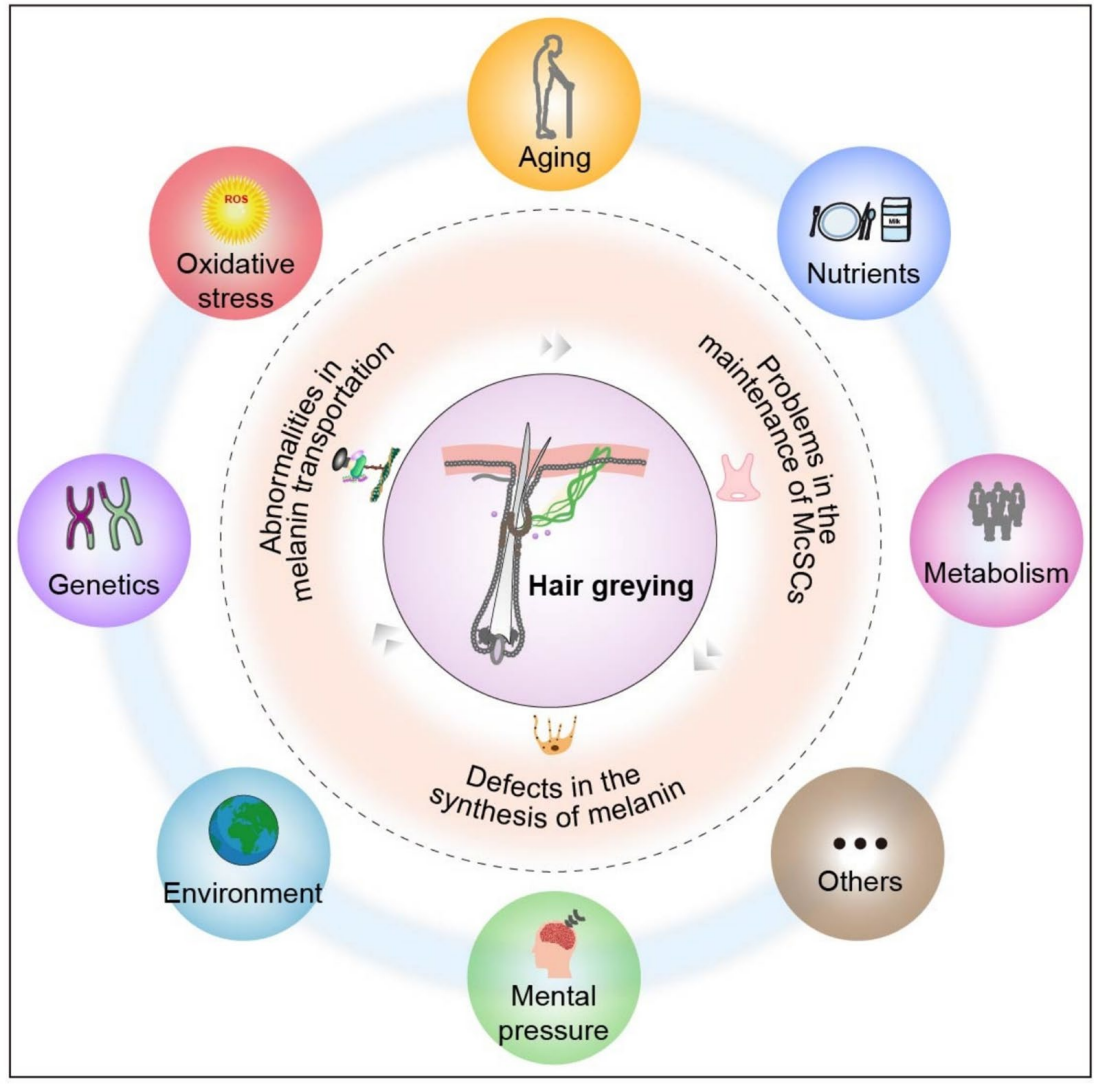
Multifaceted factors contributing to grey hair. The pathogenesis and progression of hair greying involve multiple factors, including metabolic unbalance, malnutrition, ageing, oxidative stress, genetics, environmental influences, and emotional stress. These multiple factors can be due to problems in the maintenance of McSCs, defects in the synthesis of melanin or abnormalities in melanin transportation

The reasons of occurrence and development of hair greying are multifaceted, including genetic, environmental, age-related, oxidative damage, metabolic, and other potential causes (Fig. 2). However, its critical pathogenesis remains unclear. The DNA of McSCs is damaged by environmental factors such as ionising radiation, which impair melanin synthesis and lead to unpigmented hairs [247]. Epidemiological studies have shown that the development of hair greying is also influenced by psychological and physical stress. To investigate whether psychological or physical stressors promote hair greying, Zhang et al. [270] used three methods to model stress in C57BL/6J mice with black coat colour, including restraint stress and chronic unpredictable stress. This was achieved through an injection of resiniferatoxin, an analogue of capsaicin, which showed that diverse stressors induced hair greying. Meanwhile, anxiety, depression, and other severe neuropsychiatric consequences can result from hair greying, which could produce a major negative impact on quality of life. This mood disorder and grey hair phenomenon may form a potentially vicious circle. Due to the diversity of natural products and their derivatives, we categorised them into three groups based on their mechanisms of action: regulation of the proliferation and differentiation of McSCs, promotion of melanin synthesis, and regulation and transport of melanosome.

## Regulation of proliferation and differentiation of McSCs by natural products and their derivatives

McSCs in the hair follicle are necessary for hair pigmentation, but they are tightly controlled by the hair growth cycle. During the telogen phase, McSCs remain quiescent and are located in the follicular stem cell ecological niche (bulge) and the hair germ [163]. During the early anagen growth phase, McSCs are activated by Wnt signalling, undergoing proliferation, differentiation, and migration. These are differentiated melanocytes that migrate downwards into the hair bulb to regenerate pigmented hair [180]. Meanwhile, the de-differentiated McSCs migrate to the bulge and upper outer root sheath regions, where fewer associated activator proteins are present, and then home back to the hair germ during the catagen phase for continued proliferation and differentiation to pigment the hair during the next hair growth cycle [212]. Wnt/β-catenin is essential for epithelial and melanocytes following induction of hair follicle regeneration [180]. Environmental stimuli such as ultraviolet B (UVB) irradiation can induce McSCs to accelerate their differentiation into functional melanocytes to produce visible pigment [212]. The mechanism of hyperactivation of sympathetic nerves induced by acute stress can drive the depletion of McSCs resulting in hair greying [270]. When the sympathetic nerves are activated by stress, releasing large amounts of norepinephrine, a stress hormone that binds to the β2 adrenergic receptors (β2AR) of McSCs, causing quiescent McSCs to proliferate rapidly. This is followed by their differentiation, migration, and permanent depletion from the niche [270].

**Fig. 3:**
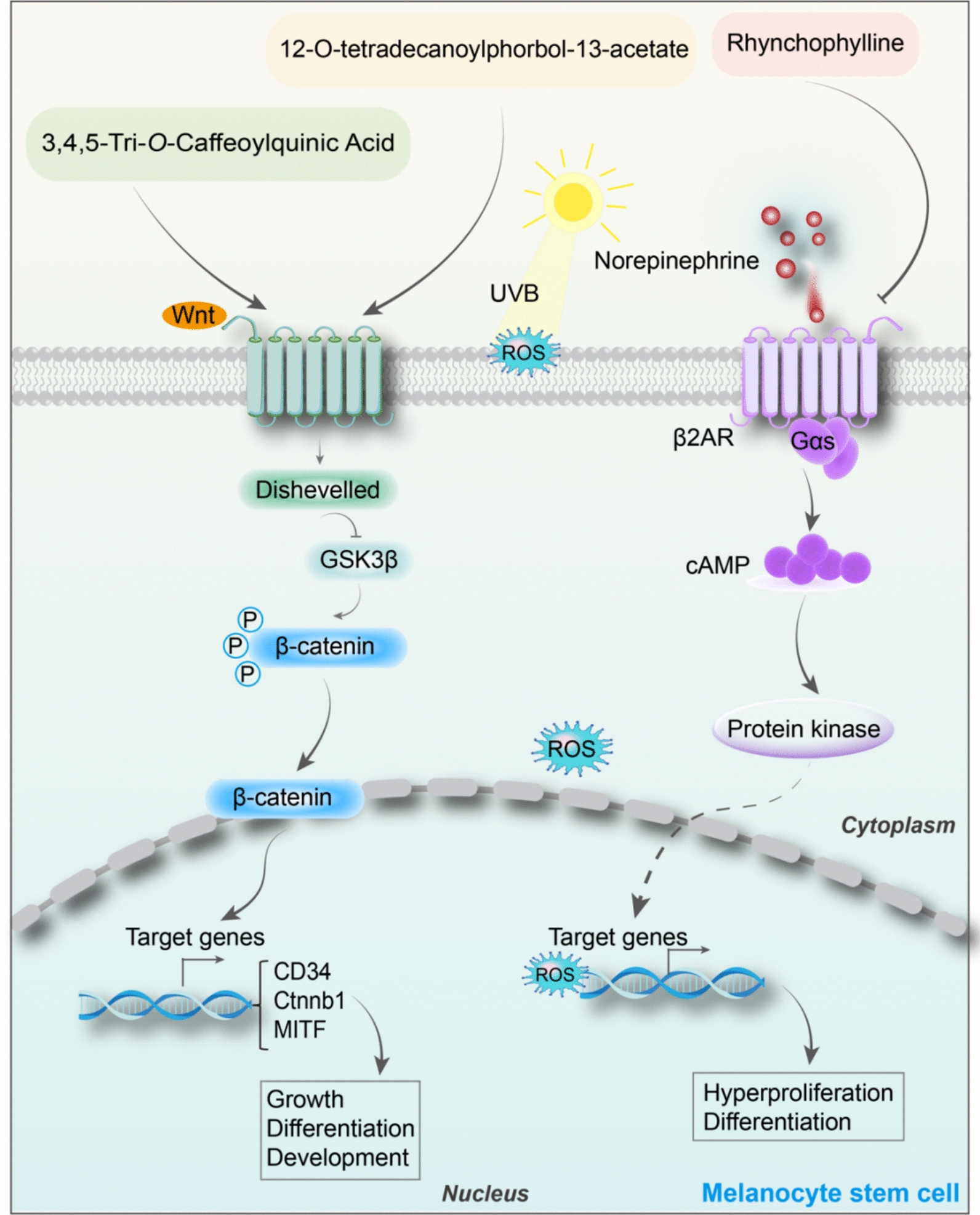
The pharmacological mechanisms of regulating abnormal proliferation and differentiation of melanocyte stem cells (McSCs) by natural products and their derivatives. Natural products and their derivatives, including 3,4,5-Tri-*O*-Caffeoylquinic acid, 12-O-tetradecanoylphorbol and Rhychophylline, regulate the hyperproliferation and differentiation of melanocyte stem cells (McSCs) via the Wnt and NE-ADRB2 signalling pathways

So far, the underlying mechanisms are not fully understood. Over the decades, there have been many studies on the regulation of McSCs proliferation and differentiation. Based on literature research, we have summarised the following natural products and their derivatives related to the regulation of McSCs proliferation and differentiation, the therapeutic effects of natural products and their derivatives have been described in detail in Fig. 3 and Supplementary materials Table 1.

### 3,4,5-tri-*O*-caffeoylquinic acid

3,4,5-tri-*O*-caffeoylquinic acid, a caffeoylquinic acid derivative, ameliorates the learning and memory deficits in ageing mouse models[192]. Previously, it was reported hair regrowth in eight-weeks-old C3H male mice and human dermal papilla cell proliferation by activating β-catenin to stimulate the initiation of the anagen phase of the hair follicle cycle [14]. Moreover, 3,4,5-tri-*O*-caffeoylquinic acid has been shown to enhance pigmentation during the anagen phase of the hair cycle in C3H mice, and human epidermal melanocytes and B16F10 murine melanoma cells by up-regulating the expression of β-catenin. It also increased expression of CD34, a marker of McSC differentiation [15, 101]. In addition, 3,4,5-tri-*O*-caffeoylquinic acid promotes hair regrowth and stimulates pigmentation via upregulating Wnt/β-catenin and Endothelin Receptor B signalling pathways in McSCs [13, 218]. These findings point to potential treatments for hair greying by regulating the proliferation and differentiation of McSCs by 3,4,5-tri-*O*-caffeoylquinic acid.

### 12-*O*-tetradecanoylphorbol-13-acetate

12-*O*-tetradecanoylphorbol-13-acetate (TPA) is a naturally occurring small molecular drug. It is involved in cell proliferation and differentiation [39, 75]. These results imply that biological processes such as proliferation, migration, and differentiation of McSCs may be regulated by TPA-mediated signalling. Qiu et al. [178] found that whether in vitro topical application of TPA to seven-weeks-old mice for four weeks or the treatment of murine melanoblasts iMC23 cells with 200 nM TPA for 6 h can stimulate the proliferation and differentiation of McSCs and their progeny for hair matrix pigmentation via activating Wnt/β-catenin signalling. This finding may provide a novel therapeutic strategy for hair pigmentation disorders.

### Rhynchophylline

A natural alkaloid, rhynchophylline is the main bioactive ingredient of *Uncaria rhynchophylla* (Miq.) Miq., and attenuates ‌lipopolysaccharide-induced pro-inflammatory responses [73, 205]. Li et al. [124] used computer-aided drug design and ZINC15 database for high-throughput screening of β2AR inhibitors and found that rhynchophylline showed a strong affinity against the receptor and obtained verification at the melanoma cell lines A2058 and B16F10. This indicates that rhynchophylline can prevent stress-induced hair greying. Therefore, rhynchophylline represents a promising natural β2AR inhibitor for preventing stress-induced hair greying.

## Promotion of melanin synthesis by natural products and their derivatives

Melanocytes are responsible for synthesising melanin, including eumelanin (brown to black) and pheomelanin (yellow to reddish-brown), and transferring it to the surrounding pre-cortical keratinocytes, and incorporating it into the growing hair shafts [210, 224]. Melanogenesis is a complex process involving a series of enzymatic and non-enzymatic reactions. The enzymatic conversion of L-tyrosine to L-DOPA and then to dopaquinone is the first step in the melanogenesis pathway [203]. A necessary element for the synthesis of pheomelanin is cysteine or reduced glutathione, which affects the pheomelanin to eumelanin ratio [4]. Generally, the synthesis of melanin is a complex process, with a multitude of genes influencing its production, such as tyrosinase-related protein 1 (*TRP-1*), glycogen synthase kinase-3 beta (*GSK3β*), *β-catenin*, cyclic adenosine monophosphate (*cAMP*), cAMP-response element binding protein (*CREB*), and solute carrier family 7 member 11 (*SLC7A11*) [93, 185, 228]. High levels of α-melanocyte-stimulating hormone (α-MSH) bind with Melanocortin 1 Receptor, resulting in the activation of adenylyl cyclase and elevation in the level of cellular cAMP, which then activates protein kinase A (PKA). This propels the catalytic subunit of PKA into the nucleus to phosphorylate CREB. When microphthalmia-associated transcription factor (MITF) is bound by active CREB, it triggers the transcription of melanogenic enzymes [12, 122, 191].

Specialised organelles called melanosomes are a prerequisite for melanin synthesis [184]. Melanosomes undergo four morphologically distinct stages [45]. The initial two stages are devoid of pigmentation but are distinguished by the presence of intraluminal proteinaceous fibrils. These commence formation in Stage I and are fully developed by Stage II. During the early stages, melanosomal structural proteins, such as pre-melanosomal protein 17 and melanoma antigen recognised by T-cells 1, are responsible for fibre formation [184]. When the fibrous striations are fully formed and elliptical, meaning that melanin synthesis has begun, enzymes such as TRP-1, TYR, and dopachrome tautomerase (DCT) are required to synthesise the phenolic amino acid precursor L-tyrosine into melanin [184]. By Stage III, melanin is deposited on top of the protofibrils, thickening and darkening them until Stage IV blurs all internal structures. Stage IV melanosomes are translocated along microtubules from the centre of the cell to the tips of actin-rich dendrites and are transported to neighboring keratinocytes [43]. Meanwhile, the general mechanism of melanin synthesis involves a complex chemical reaction in which large amounts of reactive oxygen species are generated [162]. With age, the defence function of antioxidant systems such as catalase, glutathione, and superoxide dismutase declines. An imbalance between cellular oxidative and antioxidant systems, along with accumulation of oxidative damage and downregulation of Bcl2, can reduce the anti-apoptotic capacity of melanocytes, ultimately leading to hair greying [162]. Grey hair is notably observed in Bcl2^−/−^ mice, and the expression of Bcl2 decreases. This may be one of the mechanisms of physiological hair greying in the older people and may also be a form of self-protection [162]. In the biology of hair pigmentation, after the hair follicle undergoes the 10th cycle, the ability of melanocytes to produce melanin decreases, leading to depigmented hair [225]. Repeatedly plucking hairs form mice accelerated the cycle of hair follicles and the formation of white hair [257]. Because androgenic alopecia has the characteristics of hair follicle miniaturisation and shortened hair cycle, the incidence of white hair may be higher than normal [274].

**Fig. 4:**
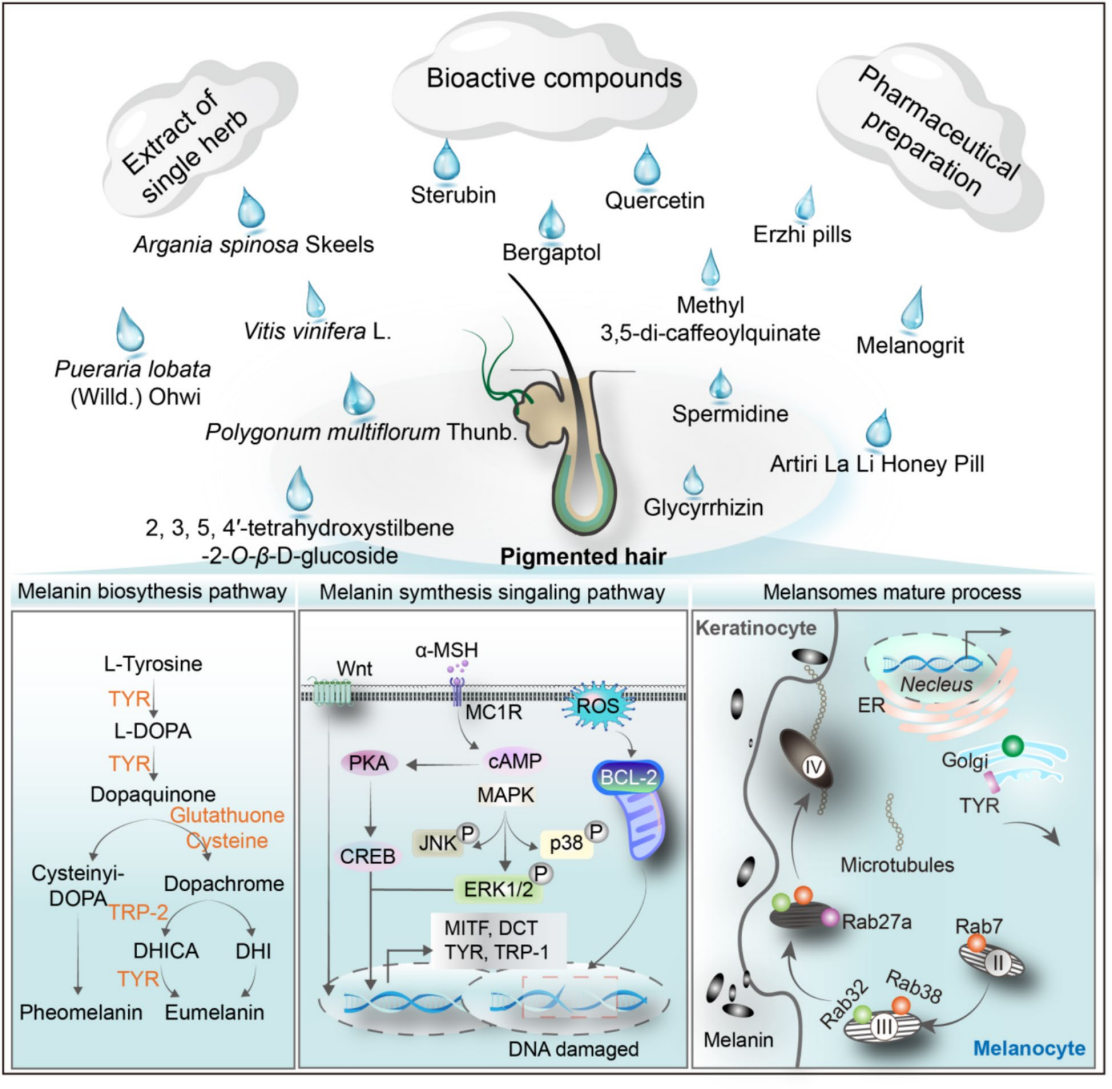
The pharmacological mechanisms of promoting melanin synthesis by natural products and their derivatives. Natural products and their derivatives, including bioactive compounds, single herb extracts, decoction, and other formulations, can promote melanin biosynthesis by regulating melanogenesis metabolic pathways, melanin biosynthesis pathways, such as PKA/CREB and MAPK signalling pathways, and melanosome maturation processes

The regulation of melanin synthesis pathways may represent a promising avenue for grey hair treatment. We reviewed the relevant literature and summarised the potential mechanisms of action of natural products and their derivatives regulating melanin synthesis (Fig. 4 and Supplementary materials Tables 2–4). These natural products and their derivatives include bioactive compounds, extracts of single herbs, and pharmaceutical preparation.

### Bioactive compounds

**Fig. 5:**
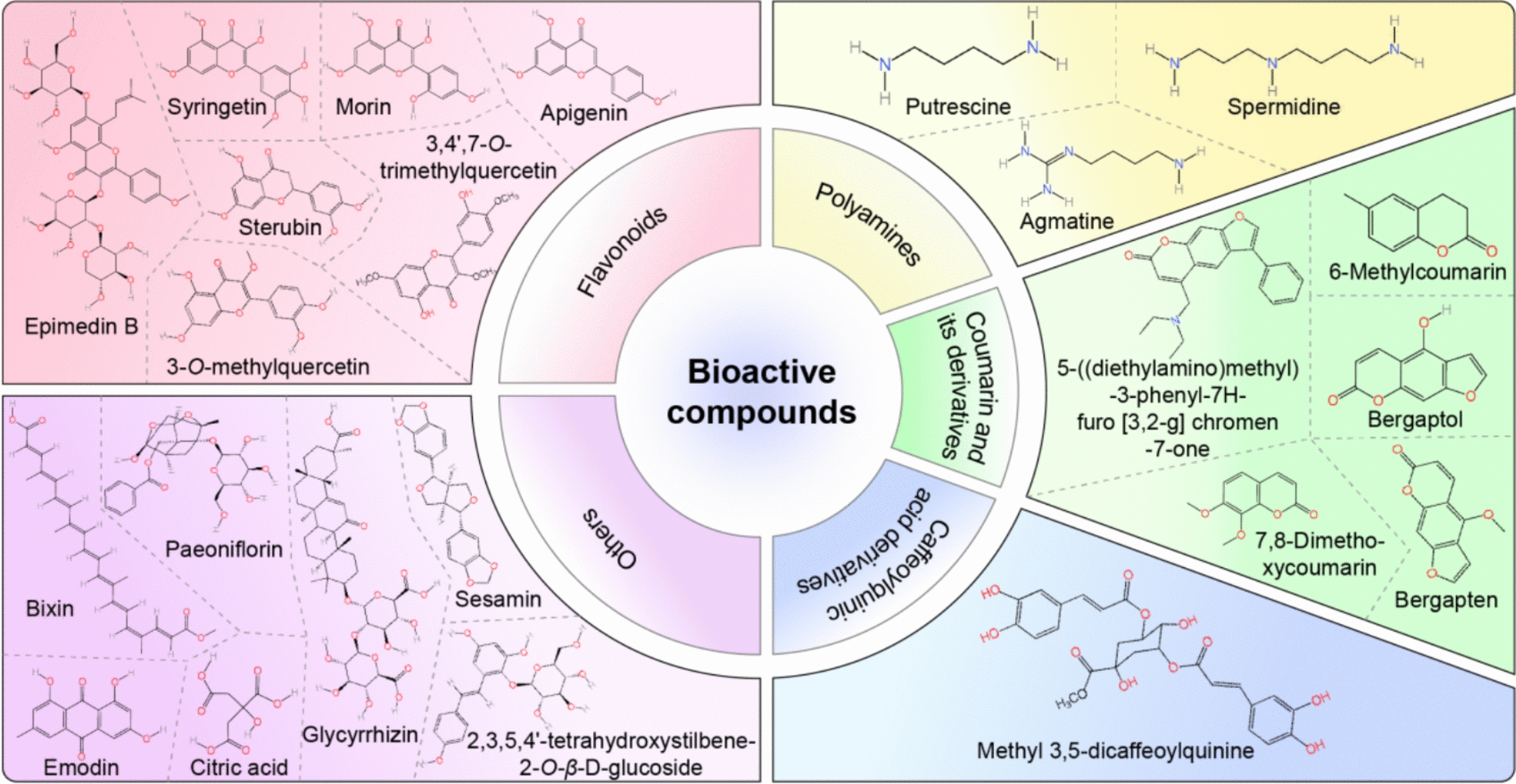
The chemical structures and classification of bioactive compounds in the treatment of hair greying. Through a literature review, we identified that bioactive compounds commonly used to promote melanogenesis include flavonoids, coumarins, and polyphenols for the treatment of hair greying

Bioactive compounds are essential compounds found in nature, including polyphenols, flavonoids, alkaloids, fatty acids, polysaccharides, vitamins, and carotenoids. These have considerable biological activity in the context of health and nutrition [19, 116, 286]. Bioactive compounds have a wealth of pharmacological activities, including anti-inflammatory [230], antioxidant [49], anti-cancer [5], and protection of intestinal immune homeostasis[287]. Bioactive compounds can promote melanogenesis [91, 164]. From a review of the literature, we found that these frequently used bioactive compounds mainly include flavonoids, coumarin, and polyphenols. The chemical structures and their classification are shown in Fig. 5.

#### Flavonoids

Widely found in plants, flavonoids have various pharmacological activities, including antioxidant [199], anti-inflammatory [149], antineoplastic[168], and lipid-lowering effects [234]. Flavonoids play an important role in melanin synthesis by affecting melanin synthesis-related proteins, especially TYR [132]. Different flavonoids show opposite effects on melanin synthesis, and this is mainly dependent on the chemical structure of the flavonoids, including possessing only hydroxyl groups at the 4′ position of the flavonoid skeleton ring B [72], 4′-*O*-methylated flavonoids, 3′ and 5′-*O*-methyl groups [72], which are beneficial in promoting pigmentary hair regeneration.

##### Sterubin

Sterubin is a natural compound that contains the highest amount of flavonoids found in Yerba Santa (*Eriodictyon angustifolium* Nutt.) [215, 217]. Sterubin, a potent inhibitor of the oxytosis/ferroptosis pathway [57], has been studied as a neuroprotective compound and a potential therapeutic for age-related neurodegenerative disease [65]. Indeed, hair greying is a hallmark of aging. Sterubin increases melanin content and TYR activity in human melanoma cell lines (HMVII) and 20 Gy X-ray-induced normal human epidermal keratinocytes (NHEKs) by activating Wnt signaling [216]. They also investigated the protective effects of topical application of 0.1 w/v% Sterubin on a grey beard hair of the volunteer for two–four weeks [216]. Although this clinical trial only had one volunteer, these findings indicate that Sterubin can be used to prevent and reduce hair greying.

##### Syringetin

An active compound present in red grapes, jambolana fruits, *Lysimachia congestiflora* Hemsl., and *Vaccinium ashei* Reade, syringetin (3,4′,5,7-tetrahydroxy-3′, 5′-dimethoxyflavone) is a flavonoid derivative [37]. It exhibits a range of pharmacological properties, including lipid-lowering [177], antineoplastic [156], osteogenic [81], and antidiabetic properties [37]. Hyunju Han and Chang-Gu Hyun provided evidence that syringetin-induced melanin production, TYR activity, and up-regulation of MITF in B16F10 cells through the activation of the PKA/CREB, PI3K/AKT, MAPK, and Wnt/β-catenin signalling pathways [72]. To assess the suitability of syringetin for topical use, they administered 3 μg/mL and 6 μg/mL concentrations of syringetin in 31 volunteers via skin patches, demonstrating that syringetin is safe [72]. Taken together, these studies have shown that syringetin may be an effective pigmentation stimulator for use in the medical treatment of hypopigmentation disorders.

##### Morin

Morin, a well-known flavanol belonging to the flavonoids class of polyphenols, is also abundantly found in various vegetables, tea, medicinal herbs, and Asian fruits [10]. Given its diverse bioactive properties, such as antidiabetic [85, 281], anti-inflammatory [24], antioxidant [8], and anticancer activities [261], morin has been extensively studied in recent years. Notably, morin has been reported to induce melanin synthesis and TYR activity by activating ERK and p38 signalling pathways in B16F10 mouse melanoma cells [201]. These findings suggest that morin could serve as a potential candidate for reducing hair greying.

##### Apigenin

Apigenin (4′,5,7-trihydroxyflavone) is a naturally occurring plant flavonoid found in various fruits and vegetables, including parsley, onions, oranges, tea, chamomile, wheat sprouts and some seasonings [172]. Owing to the abundance of bioactive components and vital nutrients, it is widely used for both nutritional and medicinal purposes [123]. Pharmacological studies have demonstrated that apigenin can reduce visceral obesity [211], alleviate liver fibrosis [176], and mitigate oxidative stress [195]. For example, Chauhan et al. [31] developed a hydroquinone-induced C57BL/6 mouse model, in which topical application of apigenin increased melanin content in hair follicles by enhancing TYR expression, and promoting melanin synthesis. Therefore, apigenin may be a promising candidate for treating pigmentation disorders.

##### Epimedin B

Epimedin B is one of the main flavonoid components of *Epimedium brevicornum* Maxim., a traditional Chinese medicainal herb. It has been reported to exhibit anti-inflammatory actibity [134], anti-pancreatic cancer effects [35], and the ability to modulate gut microbiota [121]. Epimedin B is a major component of neuroprotective ingredients [273], induces melanogenesis, and activates TYR [77]. Hong et al. [78] using a combination of in vitro and in vivo assays, have demonstrated that Epimedin B increases melanin production and promotes both melanosome biosynthesis and transport through AKT-mediated GSK3β/β-catenin, p70 S6 kinase cascade, p38/MAPK, and ERK/MAPK signalling pathways. Epimedin B can stimulate pigmentation, which provides a new strategy for preventing hair greying and treating pigmentation disorders.

##### Quercetin and its derivatives

Quercetin is a special subclass of flavonoids found in various fruits and vegetables, including tea, onions, and apples, with a name derived from the Latin word quercetum, meaning *Quercus robur* (oak) [202]. Quercetin is a potent inhibitor of TYR activity and melanogenesis in B16F10 melanoma cells [59]. However, in human melanoma cells, quercetin has the opposite effect and stimulates melanogenesis. The melanogenesis-modulating activity of quercetin has been reported to be dependent on its concentration [263]. In Japan, Kosei Yamauchi et al. have focused on quercetin derivatives, particularly 3,4′,7-*O*-trimethylquercetin and 3-*O*-methylquercetin. These compounds have been shown to increase melanin content in B16F10 cells and in a three-dimensional skin model by increasing the expression of melanin-related proteins such as MITF, TYR, and TRP-1 [258–260]. These findings suggest that quercetin and its derivatives, especially 3,4′,7-*O*-trimethylquercetin and 3-*O*-methylquercetin, could be useful products and agents in preventing hair greying.

#### Coumarin

Coumarins are a large class of phenolic compounds recently identified in plants, fungi, and bacteria. They have attracted substantial scientific interest due to their potent pharmacological activities, including improving glucose metabolism [44, 127], acting as antileishmanial agents [64], and inhibiting inflammatory factors [250]. Many drug candidates targeting the signalling pathways involved in melanin synthesis have been developed [71, 245]. Of all compounds involved in treating depigmentation disorders, coumarin derivatives, such as psoralen and bergapten have been widely used in clinical trials [9, 151]. In 1998, W McNeely reported the use of oral coumarin combined with ultraviolet A (UVA) exposure in patients with vitiligo [151]. Deeper understanding of the biological activity of coumarin and its derivatives, along with methods to improve their bioavailability, may lay a stronger foundation for their future therapeutic applications.

##### 5-((diethylamino)methyl)−3-phenyl-7H-furo [3,2-g] chromen-7-one

8-methoxypsoralen, a well-known component of furocoumarin, has been shown to promote melanogenesis in a C57BL/6 mouse model of vitiligo [90, 104]. However, due to several side effects such as skin cancer, hepatic steatosis, and hepatoxicity [102, 283], Zang et al. [268] synthesised a novel series of furocoumarin derivatives, of which 5-((diethylamino)methyl)−3-phenyl-7H-furo[3,2-g] chromen-7-one promoted melanogenesis in B16 cells. They used a hydroquinone-induced vitiligo mouse model and human epidermal melanocyte cell lines (PIG1 and PIG3V). Furocoumarin derivatives increased the number of skin basal layer melanocytes and melanin-containing hair follicles, and upregulated melanin synthesis and TYR activity by activating cAMP/PKA and MAPK signalling pathways [268]. Therefore, furocoumarin derivatives may have considerable potential as therapeutic agents for treating grey hair and pigmentation disorders.

##### 6-Methylcoumarin

6-Methylcoumarin, a type of methylcoumarin derivative, has several pharmacological properties, including anti-inflammatory [107] and anti-bacterial [182], and anti-atherosclerosis effects [154]. In B16F10 mouse melanoma cells, it stimulates melanogenesis by upregulating p-p38, p-PKA, p-CREB, p-GSK3β, and β-catenin, while downregulating p-ERK, p-AKT, and p-β-catenin [115]. Kim et al. [115] evaluated the safety for topical applications using a primary human skin irritation test on the normal skin of 31 healthy volunteers and found it to be a safe substance. These findings indicate that 6-methylcoumarin has considerable potential for use as a topical agent to prevent hair greying.

##### Bergaptol

Bergaptol is a natural furocoumarin derived from *Psoralea fructus,* that is the dried and matured fruit of *Psoralea corylifolia* L., which has anti-tumour [198], anti-inflammatory, and neuroprotective activities [243]. For thousands of years, *Psoralea fructus* has been used to treat skin diseases, especially vitiligo, with a satisfactory curative effect [42]. Recently, bergaptol has been shown to promote 1-phenyl-2-thiourea (PTU)-induced hyperpigmentation more effectively than psoralen in zebrafish larvae, Yu et al. explored the effect of bergaptol on cellular melanogenesis and found that it may increase melanin synthesis in B16F10 melanoma cells by regulating the p-P38 and p-ERK signalling pathways [265]. Therefore, bergaptol may be a feasible strategy for discovering a potential candidate drug to prevent hair greying and treat pigmentation disorders.

##### Bergapten

Bergapten is a natural furocoumarin, also known as 5-methoxypsoralen, and has a wide range of pharmacological effects, including neuroprotective, organ protective, anticancer, anti-inflammatory, antimicrobial, and antidiabetic activities [129]. Bergapten has long been used in combination with UVA irradiation to treat depigmentation disorders. However, the low content of bergapten in medicinal plants remains a limitation. To address this, Zhao et al. [279] applied a structure-based protein-engineering approach to synthesise bergapten for use in increasing hydroquinone-induced pigmentation in C57BL/6 mice. This facilitated the pigmentation in PTU-induced zebrafish embryos and elevated the melanin content in B16F10 melanoma cells by upregulating TYR expression. Bergapten is promising as a drug candidate to protect hair greying and treat pigmentation disorders, and this structure–function approach to protein engineering also provides a new and alternative strategy for treating pigmentation disorders.

##### 7,8-Dimethoxycoumarin

A natural coumarin compound present in various plants, called 7,8-dimethoxycoumarin, possesses anti-secretory, anti-inflammatory [206], antioxidant properties, and the ability to treat acute renal failure [157]. In 2020, a noteworthy study reported that 7,8-dimethoxycoumarin increased melanogenesis and TYR activity in B16F10 cells by upregulating the expression of TRP-1, tyrosinase-related protein 2 (TRP-2), and MITF, and interfered with the phosphorylation of ERK in the MAPK pathway [120]. Therefore, 7,8-dimethoxycoumarin may be a viable option for potential melanin-producing activator and anti-grey hair applications.

#### Caffeoylquinic acid

Caffeoylquinic acid, a member of the phenolic acid family of polyphenols, is commonly found in tea, wine, and coffee. It is a widely used bioactive compound with substantial therapeutic and biological activity [155], including anti-inflammatory [246, 248], gut microbiota-regulating [139], anti-neuroinflammatory [141], and antioxidant effects[89], and is used to treat various diseases. Pharmacological activity analysis indicated that caffeoylquinic acid and its derivatives positively affected pigment-related diseases such as vitiligo and hair greying. Methyl 3,5-di-caffeoylquinate is a type of caffeoylquinic acid derivative isolated from the stems and leaves of *Erigeron annuus* (L.) Desf [110]. Caffeoylquinic acid derivatives have a wide range of biological activities. Kim et al. [110] found that methyl 3,5-dicaffeoylquinine increased TYR activity and melanin production in B16F10 through a master regulator of melanocyte activity, MITF, and methyl 3,5-dicaffeoylquinine-mediated MITF activation relies on cAMP/PKA signalling and p38 MAPK phosphorylation. The melanogenic effect of methyl 3,5-dicaffeoylquinine was confirmed by assessing increased levels of melanin production in normal human epidermal melanocytes and human melanoma cells. These findings imply that future research and applications should consider the screening and development of natural drugs for pigmentation disorders, including hair greying and depigmented skin disorders.

#### Polyamines

Natural polyamines, including putrescine, spermidine, and norspermidine, are commonly found in traditional Chinese herbs, insects, bacteria, and marine sponges [140]. A substantial body of research has confirmed the positive effect of polyamines on human health, including potential benefits in cancer [256], age-related diseases [70], and neurodegenerative diseases [188]. In addition to these pharmacological effects, polyamines can induce TYR activity to promote pigmentation [208]. The specific pharmacological effects and associated mechanisms are detailed in Supplementary materials Table 2.

##### Putrescine

Putrescine is the smallest member of the polyamine family and is abundant in living mammalian cells. Putrescine promoted pigmentation in human skin explants and primary normal human epidermal melanocytes by inducing TYR, the rate-limiting enzyme in melanin synthesis [208]. These findings highlight putrescine as a regulator of pigmentation and provide new avenues for developing novel treatments for pigmentation disorders. Natchanok Talapphet and Moon-Moo Kim have confirmed that putrescine enhances melanogenesis in H_2_O_2_-induced B16F1 cells by increasing the expression of *MITF*, *TYR*, *TRP-1*, and *TRP-2* genes [219]. Therefore, putrescine may serve as a natural compound with protective potential against premature hair greying.

##### Spermidine

Spermidine is a polyamine naturally present in living organisms and is increasingly recognised as an inhibitor of ageing and oxidation. A randomised clinical trial has shown that supplementation with spermidine results in a beneficial effect on memory function or other neuropsychological, behavioural, or physiological parameters [193]. Brito et al. [22] have found that spermidine can promote melanogenesis in B16F10 cells, human melanoma MNT-1 cells, and primary human melanocytes MNT-1 by improving the stability of TRP-1 and TRP-2. Meanwhile, spermidine has been shown to increase the amount of melanin in the body through a human skin equivalent model. Therefore, spermidine is a promising natural compound for addressing hair greying.

##### Agmatine

An endogenous polyamine derived from L-arginine [190], agmatine (a.k.a. 1-(4-aminobutyl) guanidine) is famous for its role in preventing from neurological diseases [108, 173]. Only a tiny fraction of endogenous agmatine originates from cellular enzymatic de novo synthesis, it is also found in an anti-ageing plant called Manaca [117]. Eun-Jeong Kwon and Moon-Moo Kim found that agmatine could increase melanin synthesis and TYR activity by regulating the MITF transcription factor via the BMP-6/p38 signalling pathway in B16F1 cells [117]. These findings suggest that agmatine may be a potentially effective drug ingredient against premature hair greying.

#### Others

In addition to the above-mentioned compounds, many other bioactive molecules can improve depigmentation diseases. These include bixin, gambogic amide, and citric acid. Information on some other active natural products and derivatives has been summarised in Supplementary materials Table 2.

##### Bixin

There has been an increasing focus on bixin, an FDA-approved natural food colourant from the seeds of the achiote tree (*Bixa orellana* L.) native to tropical America [209, 221, 227]. Bixin has a wealth of pharmacological activities, including anti-inflammatory [266], antioxidant[204], anti-fibrosis[143], and skin protection [221]. Systemic administration of the bixin protects skin against solar UV-induced damage through activation of nuclear factor erythroid 2 (E2)-related factor 2 (NRF2) [221]. Likewise, de la Vega et al. [48] have found that bixin protects human primary epidermal melanocytes from H_2_O_2_-induced loss of viability through activation of NRF2. Topical use of bixin also protected psoralen UVA-induced hair greying in C57BL/6J mice by upregulating NRF2. Therefore, the method of using bixin topically may be a potential new way to treat pigmentation disorders.

##### *Cistanche deserticola* polysaccharide

*Cistanche deserticola* polysaccharide is a major active component isolated from the fleshy stems of *Cistanche deserticola* Ma. It possesses various pharmacological activities, including neuroprotective effects [96], immune regulation [56], gut microbiota regulation [137], and as an antioxidant [80]. *Cistanche deserticola* polysaccharide has been shown to promote melanogenesis in human epidermal melanocytes and mouse melanoma B16F10 cells by activating the MAPK signalling pathway. It also enhances the NRF2/HO-1 antioxidant pathway, thereby protecting melanocytes from oxidative stress-induced damage [84]. Thus, *Cistanche deserticola* polysaccharide may be a novel drug for preventing hair greying and treating pigmentation disorders.

##### Citric acid

Citric acid (2-hydroxy-1,2,3-propane-tricarboxylic acid) is a weak organic acid commonly found in citrus fruits, such as lemon, grapefruit, tangerine, and orange [285]. It has enhanced intestinal immunity [83], anti-bacterial [158] and antihypertensive activities [159]. Citrate plays a critical role in intramelanosomal pH in the regulation of melanogenesis [18]. Citric acid promotes melanin synthesis in B16F10 cells by regulating the activity of TYR. However, it inhibits melanin synthesis in HMV-II human melanoma cells and human epidermal melanocytes [285]. This opposing regulatory effect is due to the different tolerances of human and mouse cells to citric acid. Nevertheless, further research is required to elucidate and substantiate this hypothesis.

##### Paeoniflorin

Paeoniflorin is the major active component of the total glycoside of paeony, which is extracted from the dried root of *Paeonia lactiflora* Pall. Due to the diversity of its pharmacological activities, including anti-inflammatory[254], immunoregulatory[272], and anti-depressive effects[238], paeoniflorin is widely used in the treatment of various diseases. *Paeonia lactiflora* Pall. has been used to treat vitiligo for thousands of years in traditional Chinese medicine (TCM). Paeoniflorin significantly increased melanin content and intracellular TYR activity of human melanocytes by regulating the ERK/CREB pathway, and improved monobenzone-induced vitiligo in mice [82]. The results of these studies provide compelling evidence for the efficacy of paeoniflorin in depigmentation treatment.

##### Glycyrrhizin

The major water-soluble ingredient isolated from *Glycyrrhiza glabra* L., a native plant of Central and South-Western Asia, glycyrrhizinis has shown strong anti-inflammatory [239], anti-depressive [232], amd neuroprotective effects [55]. Approximately 20 years ago, glycyrrhizin was shown to stimulate melanogenesis in B16F10 cells through cAMP signalling, which was further confirmed by using an inhibitor of protein kinase A (H-89) [119]. Therefore, glycyrrhizin may be a new strategy for treating pigment deficiency-related diseases.

##### Emodin

Emodin is a bioactive compound and is the main ingredient in *Rheum palmatum* L. Recent pharmacological studies suggest that emodin may be a valuable therapeutic option for treating various diseases, including cardiac hypertrophy [61], anti-fibrotic [181], and breast cancer [135]. Emodin is also derived from *Polygonum multiflorum* Thunb.. More than 2000 years ago, documented records stated that *Polygonum multiflorum* affected hair pigmentation. Emodin promotes melanin synthesis in B16F1 cells by activating MITF transcription factors to increase the expression of TYR, TRP-1, and TRP-2 [111]. Therefore, emodin may become a potential drug to prevent hair greying, but its safety requires additional assessment.

##### Olive-derived phenolic compounds

Olive leaves are rich in phenolic compounds, including oleuropein, oleocanthal, and oleacein. Generally, they are as predominantly consumed as a traditional diet [29]. However, they have also been used as drugs to improve chronic diseases such as cardiovascular diseases [54], osteoporosis [63], and lipid metabolism disturbance [146]. They are also used in dermatology, particularly in repairing the natural skin-barrier function [229]. Olive-derived phenolic compounds impact skin protection by increasing the gene and protein expression of MITF, TYR, TRP-1, and DCT to promote melanin production in human epidermal melanocytes and B16F10 mouse cells [38]. These findings underscore the potential of olive-derived secoiridoids as active agents for treating hair greying.

##### Sesamin

Sesamin is the main functional compound of *Sesamum indicum* seeds and sesame oil. Like olives, not only are sesame seeds delicious, but they can also be used as a health-promoting agents, for example, attenuating inflammatory responses [105], protecting against colitis [32], and ameliorating lung injuries [186]. During the screening programme for the development of pigmenting agents, Jiang et al. [98] have reported that sesamin induces melanogenesis in B16 melanoma cells through activating cAMP signalling to up-regulate the expression of MITF and TYR and suggested it might be useful as a sunscreen. Therefore, sesamin could be used as a food supplement for hair pigmentation.

##### 2,3,5,4′-tetrahydroxystilbene-2-*O*-*β*-D-glucoside

The water-soluble active component extracted from dried tuber root of *Polygonum multiflorum*, 2,3,5,4**′**-tetrahydroxystilbene-2-*O*-*β*-D-glucoside (TSG) has anti-depression [126], anti-fibrosis[87], anti-ageing [60], and neuroprotective activities [36, 153]. TSG is a potent TYR activator and a melanogenesis stimulator [66]. Jiang et al. [97] demonstrated the potential efficacy of TSG as a natural treatment for hair greying and found that it could increase melanin content and TYR activity in B16 cells by inducing sustained MITF upregulation and activation of the cAMP/CREB pathway. Furthermore, using the p38 MAPK inhibitor (SB203580), it was shown that TSG can promote melanin synthesis through MAPK activation. Although the liver injury caused by *Polygonum multiflorum* has received attention, our previous studies have shown that the liver injury caused by *Polygonum multiflorum* is specific, and its main pharmacodynamic component, TSG, is safe and effective [271]. Therefore, these studies suggest that TSG may be used to prevent hair greying.

### Extract of single herb

**Fig. 6:**
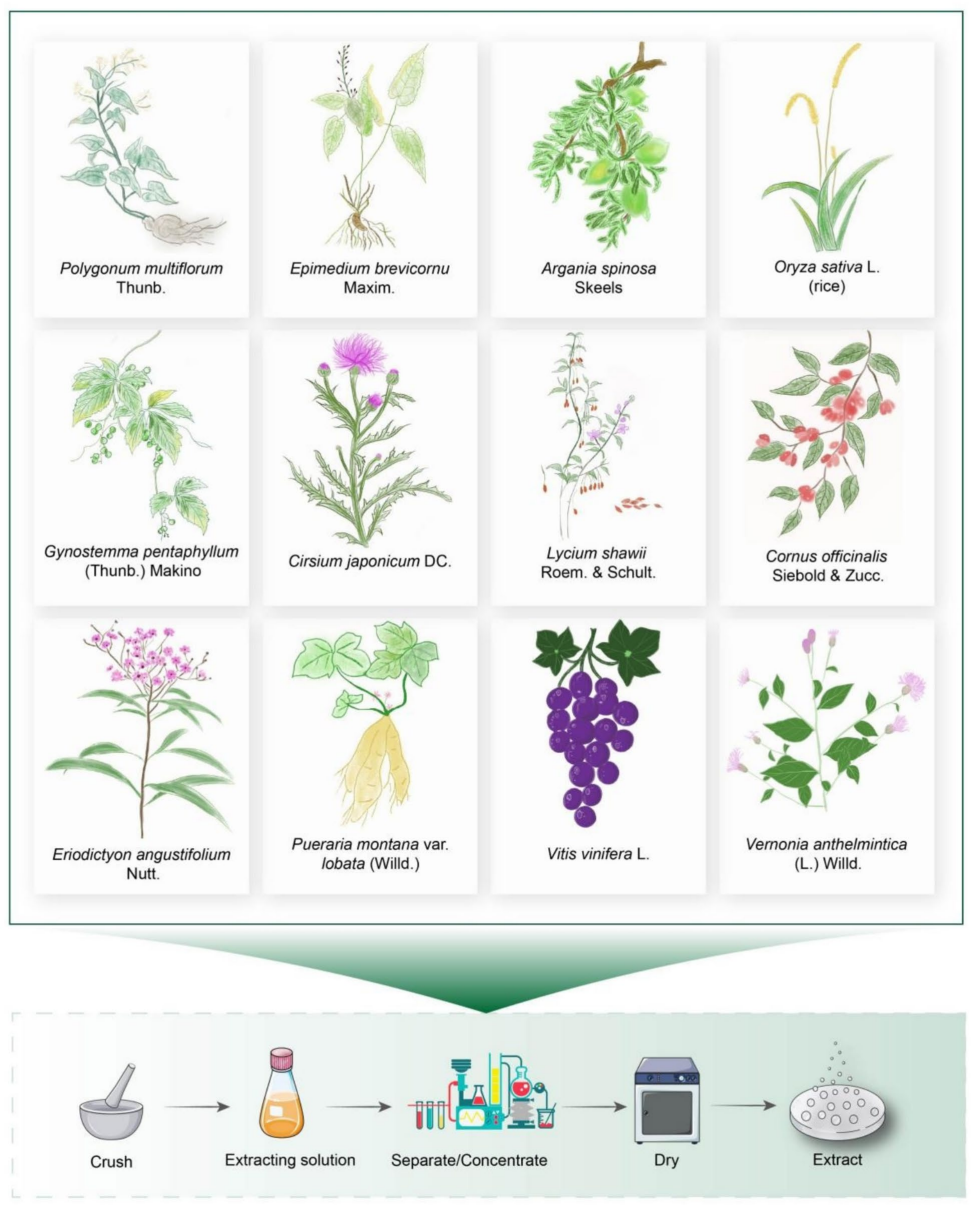
Schematic diagram of single herb extracts for the treatment of hair greying. In this review, we have summarised 12 single herb extracts that treat hair greying by promoting melanogenesis

The use of single herbs dates to ancient civilisations, especially in China. Various plants have been used to treat different diseases, including hair greying. Medicinal plants and herbs could provide more efficacious treatment options with fewer side effects and they act on different targets or pathways compared to bioactive compounds. Generally, a plant is extracted by one of two methods: one ‘natural products’ method and ‘traditional Chinese medicine method’ [94]. Effects and mechanisms of various extracts of single herbs on hair greying treatment from existing studies have been summarised in Fig. 6 and Supplementary materials Table 3.

#### Polygonum multiflorum

Polygoni multiflori radix (PM, He-Shou-Wu in Chinese) is the dried tuberous root of *Polygonum multiflorum* Thunb. The *Polygonaceae* plant has been widely used in China for more than one thousand years [237]. In traditional clinical use, it is a well-known tonic herb, which has beneficial effect of treating a deficiency of essence and blood, dizziness, and premature whitening of hair [222]. Modern pharmacological studies have shown that PM works as a defence against oxidative stress-driven degenerative diseases, age-related changes, and inflammation-associated disorders [130]. Our previous research has shown that natural derivatives from PM as powerful rejuvenators for ageing [60]. As early as ten years ago, P. Sextius reported that PM extract protects in vitro primary human foreskin melanocytes from the deleterious effects of H_2_O_2_ exposure and improves pigmentation within ex vivo human hair follicles [194]. These analyses have provided a key strategy for preventing hair greying.

#### *Argania spinosa*

*Argania spinosa* fruit shell extract is derived from an endemic tree species in the western Mediterranean region, which is well-known for its nutritional and therapeutic benefits [30]. It has anti-inflammatory and antioxidant activities [147]. Proanthocyanidins, rhizopodophyllin, epicatechin, rutin, and other active ingredients are found in its hulls. Of these, rhizopodophyllin has been reported to play an important role in regulating melanogenesis [103]. Either *Argania spinosa* oil, argan press-cake, or argan fruit shell can promote melanogenesis in B16F10 cells by upregulating the expression of the melanogenic enzymes TYR, TRP-1, and DCT mRNA through the cAMP-MITF signalling pathway [148]. At the same time, To overcome the physicochemical limitations of natural compounds and their undesirable pharmacokinetic properties such as poor absorption, distribution, metabolism, and excretion in vivo, the design and application of nanocarriers has attracted extensive attention leading to nanocarrier-based drug delivery systems. Nanotechnology is an effective alternative route to improve the drug delivery and efficacy of bioactive compounds [86]. This is due to the ability of the nanocarriers to precisely control drug release, ensuring stability and activity until it reaches the target site. This substantially reduces the frequency of administration and side effects [58]. A novel nanoemulgel, formulated using microfibrillated cellulose derived from *Argania spinosa* shell and a*rgan* shell or a*rgan* press cake extracts, acts as a natural emulsifiers and increases melanin content successfully when compared to untreated B16F10 cells [21]. These related studies suggest that *Argania spinosa* could be used as a cosmetic or as a therapy aimed at increasing the production of melanin.

#### Gynostemma pentaphyllum

Gynostemma [*Gynostemma pentaphyllum* (Thunb.) Makino] is a herbaceous climbing plant form the Cucurbitaceae and Gynostemma genus. It is also named Jiao-Gu-Lan in Chinese and has a long history as a food and dairy supplement, such as a functional food, sweetener, and herbal tea [241, 244]. It has versatile pharmacological effects, including antioxidant, anti-ageing [128], gut microbiota regulation [251], anti-obesity [255], antitumour [125], and antidepressant properties [40], which are believed to confer medicinal benefits. *Gynostemma pentaphyllum* (Thunb.) Makino extract stimulates hair regrowth by acting on the transforming growth factor-β1 and Wnt signalling pathways and enhances melanogenesis in B16F10 cells induced by norepinephrine stress by increasing the expression of MITF, TYR, TRP-1, and TRP-2 [136]. These studies provide supporting preclinical evidence for the potential of Gynostemma as an anti-greying agent.

#### *Cirsium japonicum*

*Cirsium japonicum* is the dry above-ground part of th*e Cirsium japonicum* DC. (Asteraceae) in the chrysanthemum family. It is an edible and medicinal plant with anti-inflammatory [175], hepatoprotective [144], and neuroprotective properties [114]. Kim et al. [113] investigated the effects of *Cirsium japonicum* flower extract on enhancing the melanogenesis of melanocytes using the reconstituted three-dimensional skin model and ex vivo hair follicles. Treatment with *Cirsium japonicum* flower extract promoted melanogenesis in human melanocytes by upregulating the expression of MITF and TYR through cAMP signaling [113]. Therefore, the *Cirsium japonicum* flower may be used as a potential melanogenesis stimulator for hypopigmentation disorders, such as grey hair.

#### *Lycium shawii*

*Lycium shawii*, also known as Saudi Arabian Seabuckthorn, is a thorny shrub that is a member of the *Solanaceae* family [46]. *Lycium shawii* has a wide range of biological activities, such as anti-infertility [160], antimicrobial [3], and antioxidant properties [46]. In 2023, Alghamdi et al. [2] found that *Lycium shawii* methanolic extract could stimulate the proliferation and migration of the primary culture of the human melanocytes, exhibit stimulatory effects on melanosome formation and maturation, and upregulate melanogenesis-related proteins, including MITF, TRP-1, and TRP-2. This study not only hints at a potential natural medicine to treat vitiligo, but also provides new insights into the treatment of hair pigment disorders.

#### *Eriodictyon angustifolium*

Yerba Santa, commonly known as *Eriodictyon angustifolium* Nutt., is found on the west coast of the United States of America and Mexico [231]. It has a wide range of medicinal properties, such as anti-asthmatic [231], anti-inflammatory [231], and neuroprotective activities [57]. Both *Eriodictyon angustifolium* extract and dietary *Eriodictyon angustifolium* tea could reduce human hair greying [215, 217]. *Eriodictyon angustifolium* extract increases melanin synthesis in melanocytes by activating the WNT/MITF/TYR-signalling pathway, inhibiting radiation-induced DNA damage and cell death in normal human epithelial keratinocytes, and preventing and alleviating human beard and hair greying [215, 217]. Dietary *Eriodictyon angustifolium* tea showed an important protective effect against X-ray-induced hair greying in vitro [215, 217]. These results suggest that *Eriodictyon angustifolium* is a potentially effective means of combating hair greying, whether used as a herb or as a dietary supplement.

#### Pueraria lobata

Puerariae lobatae radix is the dried root of *Pueraria Lobata* (Willd.) Ohwi from the family *Fabaceae* and is commonly used in medicine or functional foods [27]. Puerariae lobatae radix regulates obesity [150], inhibits alcohol metabolism [246], and protects nerves [187]. A randomised, double-blind, placebo-controlled clinical trial has shown that topical application of *Pueraria lobata* extract for twenty-four weeks can prevent the growth of new grey hair without any significant side effects[99]. The extract of *Pueraria thunbergiana* (also belongs to *Pueraria lobata* (Willd.) Ohwi) enhances content of melanin in melan-A cells through cAMP/MITF-M signalling pathway, stimulates melanogenesis in PTU-washed zebrafish, and increases the hair melanin content significantly via topical treatment in MITF^vit/vit^ mice [170]. These findings suggest that Puerariae lobatae radix prevents follicular depigmentation by stimulating melanin synthesis.

#### *Vitis vinifera*

Grape (*Vitis vinifera* L.) is a vine of the genus Vitis in the Vitis family that is widely cultivated worldwide, including in Spain, Chile, Brazil, China, and the United States [161, 214]. It has many pharmacological effects such as anti-oxidation [207], anti-inflammatory [23], and anti-viral properties [16]. Grape extract promotes melanogenesis in B16F10 melanoma cells in the presence of α-MSH by upregulating the expression of genes related to melanin synthesis, such as *TYR, TRP-1, TRP-2,* and *MITF.* However, it exhibits minimal influence on melanin synthesis when administered independently [284]. These results have prompted the formulation of new questions and the delineation of prospective avenues for future research on depigmentation of hair.

#### *Oryza sativa*

One of the most significant and widely utilized by-products of rice (*Pueraria Montana var. lobata* (Willd.))—the most consumed grain worldwide—is rice bran [169]. The rice bran oil extract of the Thai varieties ‘Bue Bang 3CMU’ and ‘Bue Bang 4CMU’ is rich in phenolic compounds and has antioxidant properties, which can be used as an antioxidant to protect cells from peroxide damage [109, 242]. Ruksiriwanich et al.[189] showed that the ability of rice bran oil to promote melanin synthesis in B16F10 cells may be related to iron, zinc, and unsaturated fatty acids. Rice bran oil can also improve nitric oxide-induced melanin production, which demonstrates the medicinal potential of rice by-products in hair recolour.

#### *Cornus officinalis*

Cornus (*Cornus officinalis* Siebold & Zucc.) is a herb and food plant native to East Asia [53]. Modern phytochemical studies have pointed to the systematic isolation and identification of iridoids, organic acids, lignans, flavonoids, phenylpropanoids, and other active components from Cornus [62]. It is widely used in the treatment of liver and kidney diseases, diseases of the reproductive system, and weakness [62]. Modern pharmacological studies have indicated that Cornus has antioxidant [236], antidiabetic [74], anti-hepatic fibrosis [6], neuroprotection [26], and cardiovascular protection [276]. The methanolic extract of Cornus increased TYR activity to induce melanogenesis and upregulated the expression levels of MITF, TRP-1, and TRP-2 [7]. Therefore, Cornus can be used as a natural pigmentation agent to prevent hair greying.

#### *Vernonia anthelmintica*

V*ernonia anthelmintica* (L.) Willd. is an annual herb in the *Asteraceae* family and is mainly used for the treatment of vitiligo[51]. It is closely related to various bioactive compounds, including butin, caffeic acid, and luteolin. Previous studies have shown that oral administration of butin for 40 days significantly increase the number of melanin-containing hair follicles, basal melanocytes, and melanin-containing epidermal cells in the shaved skin areas of mice with hydroquinone-induced vitiligo [90]. Similarly, caffeic acid and luteolin could stimulate melanogenesis in zebrafish [118]. The combination model of the above three compounds (butin:caffeic acid: luteolin = 1:4:10) was established using a combination of pharmacological and statistical techniques to promote melanogenesis in B16F10 cells in vitro, and promote melanogenesis in zebrafish in vivo through the expression of melanin-related genes *TYR, MITF, KIT* and *DCT* [118]. Collectively, this study forms the basis for future research on hair unpigmentation.

### Pharmaceutical preparations

The function of pharmaceutical preparations is based on diverse actions of natural products, which are usually boiled and administered as a pharmaceutical preparation or tea. In recent years, the clinical research of TCM compounds has garnered increased international attention. In 2024, Jianwen Guo et al. showed a randomised, placebo-controlled, double-blind, clinical trial of the Chinese herbal compound FYTF-919 in the treatment of acute cerebral haemorrhage in the Lancet [68]. This breakthrough marks the gradual emergence of TCM compounds in international mainstream medical research, showing their potential and importance in the treatment of major diseases. More than 2,000 years ago, pharmaceutical preparations have shown advantages in treating white hair (Supplementary materials Table 5).

#### Artiri La Li Honey Pill

Artiri La Li Honey Pill (consisting of *Carum carvi* L., *Anacyclus pyrethrum* DC., *Operculina turpethum* (L.) Silva Manso, and *Zingiber officinale* Roscoe) is a traditional and clinically used pill in Xinjiang, China, which has been used for treating vitiligo for more than 20 years [282]. In hydroquinone-induced vitiligo mice and H_2_O_2_-induced vitiligo guinea pigs, Artiri La Li Honey Pill increased the number of melanin-containing hair follicles and the epidermal melanin content in the skin of vitiligo animals by enhancing the content of TYR. The monomer components, including carvone, luteolin, bakuchiol, and psoralen, significantly promoted melanin production in zebrafish [282]. In contrast, chlorogenic acid, apigenin, and betulin had no significant effect on melanogenesis in zebrafish [282]. These results suggest that Artiri La Li Honey Pill is a potentially effective clinical agent for the improvement of hair greying.

#### Er-zhi pills

Er-zhi pills, a TCM formula compose of *Herba Epimedii* and *Fructus Ligustri Lucidi* in equal proportions, is prescribed for the treatment of kidney deficiency, such as osteoporosis, post-menopausal symptoms, and menstrual disorders [262, 289]. Er-zhi pills are also regarded as a classic prescription for vitiligo [79]. Modern pharmacological studies have shown that Er-zhi pills can increase melanin leves and TYR activity in both zebrafish depigmentation model and B16F10 cells by activating the cAMP/PKA signalling pathway [79]. These findings not only support the efficacy of Er-zhi pills in treating vitiligo but have also prompted researchers to consider whether they could be used to treat bleached hair.

#### Melanogrit

Melanogrit (including *Psoralea corylifolia* L., *Acacia catechu* Wight & Arn., *Rubia cordifolia* L., and *Cassia fistula* L.) is a trademarked herbal formulation manufactured and marketed by Divya Pharmacy, Haridwar, India. The product has been approved for clinical use by the relevant regulatory agencies, as evidenced by the manufacturing licence number Uttra.Ayu-67/2005 [11]. Balkrishna et al. [11] demonstrated that melanogrit has the potential to stimulate melanogenesis in HaCat cells co-cultured with B16F10 cells by up-regulating the expression of melanin-related genes such as *MITF, TYR,* and *TRP-1*. This novel study proposes melanogrit as an effective therapy for depigmentation disorders.

## Melanosome transport and regulation by natural products and their derivatives

**Fig. 7:**
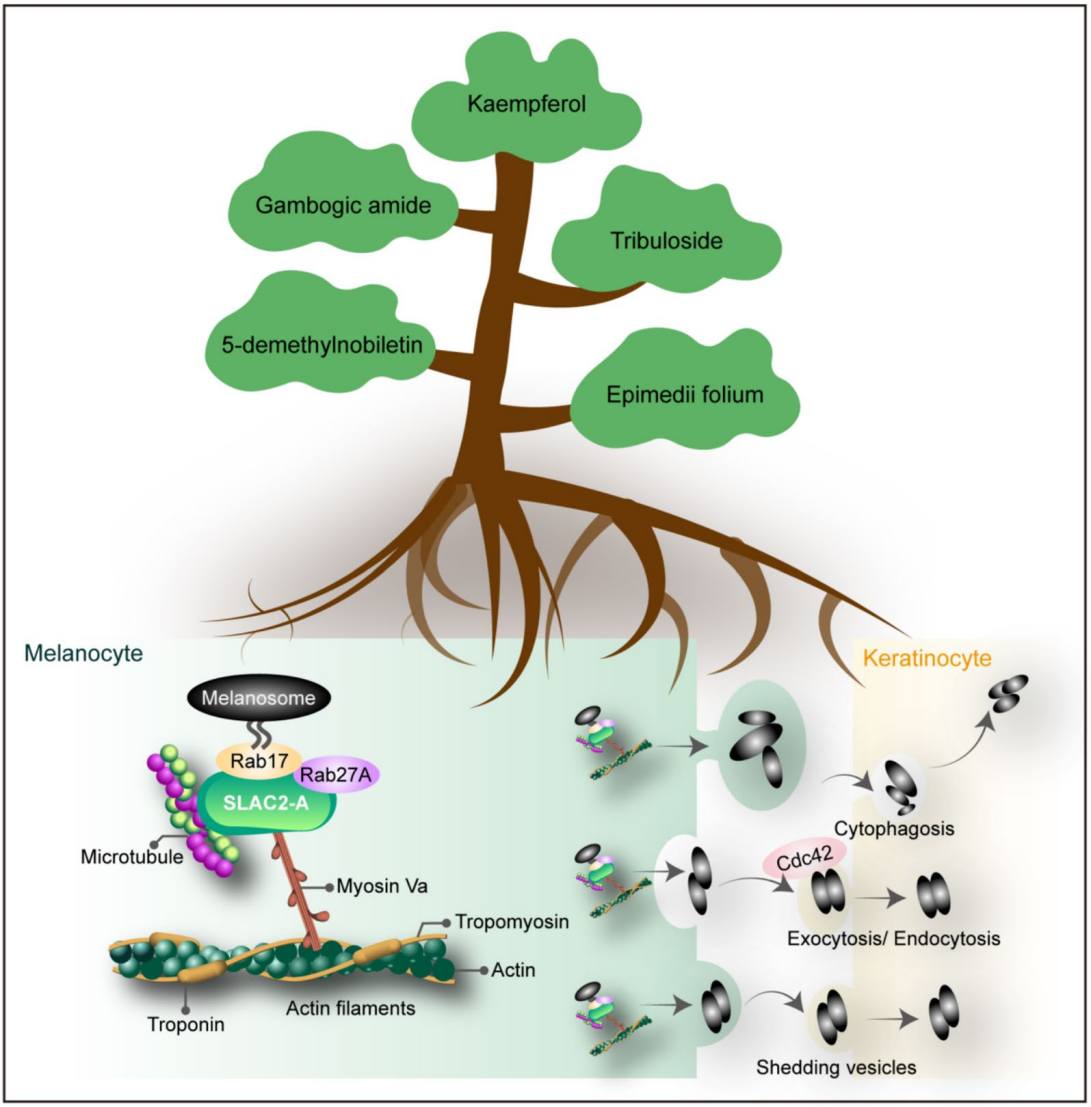
The pharmacological mechanisms of transporting and modulating melanosome by natural products and their derivatives. There are five natural products and their derivatives, including 5-demethylnobiletin, gambogic amide, tribuloside, kaempferol, and Epimedii folium can ameliorate grey hair by promoting melanosome transport

In the hair follicle, melanin is transferred to keratinocytes via dendrites to pigment newly growing hair. The underlying molecular mechanisms of melanosome transport can be classified into three categories: membrane transport, cytoskeleton-dependent melanosome transport, and transfer of melanosomes to keratinocytes [223]. Rab GTPases are integral to the regulation of intracellular membrane traffic [88]. The process of transporting pigmented melanosomes to the tip of melanocyte dendrites is dependent on microtubules and F-actin, including the actin-based motor protein Myosin Va (Myo Va) [226]. The modes of melanosome transfer from melanocyte to keratinocyte are mainly via cytophagosis, shedding vesicles, exocytosis, and endocytosis [43]. Melanocytes are protected from ultraviolet radiation, but they are likely to undergo exceptional alterations while undergoing senescence [106]. A recent study highlighted that in vitro senescent melanocytes are characterised by melanosome transport dysfunction resulting in melanin accumulation, rather than enhanced melanin synthesis [171]. As is well known, Ras-related protein Rab-27AA (Rab27), Ras-related protein Rab-17 (Rab17), and cell division control protein 42 (Cdc42) belonging to Rho GTPase family are closely involved in the regulation of intracellular vesicle trafficking due to their roles in endocytosis [165]. Therefore, the processes of dendritic growth and melanin body transport in melanocytes are of critical importance in the intricate regulation of pigmentation. We reviewed relevant literature to screen natural products and their derivatives for the prevention and treatment of white hair based on melanosome transport and regulation (Fig. 7 and Supplementary materials Table 3).

### 5-Demethylnobiletin

5-Demethylnobiletin is a hydroxylated polymethoxyflavone from citrus plants, which is a promising bioactive substance. It alleviates antibiotic-related gut damage [269], attenuates dextran sodium sulphate-induced colitis in mice by suppressing the immune response and modulating the gut microbiota [252], and provides relief from psoriasis [240]. Numerous pharmacological studies have demonstrated its anticancer [131], anti-inflammatory [50], and neuroprotective activities [267]. In addition, 5-demethylnobiletin can stimulate dendritic structure formation, melanin synthesis, and melanosome transport in B16F10 melanoma cells by activating the cAMP/CREB pathway [233]. Therefore, 5-demethylnobiletin is a promising natural agent for managing pigmentation disorders and treating hair greying.

### Tribuloside

A natural flavonoid extracted from the traditional Chinese herb *Tribulus terrestris* L., tribuloside, was initially documented in Shen-Nong-Ben-Cao-Jing and has been extensively used by traditional doctors for numerous medicinal purposes, such as type 2 diabetes [152], male sexual function [174], and breast cancer [278]. Specifically, Cao et al. [28] found that tribuloside had an impact on the production of melanin in melanocytes, zebrafish, and UVB-induced young human foreskins. Tribuloside can facilitate melanogenesis, melanocyte dendricity, and melanin transport by phosphodiesterase (PDE)/cAMP/PKA pathway [28]. Therefore, tribuloside could be a promising and effective natural product for restoring hair pigment.

### Kaempferol

Kaempferol is a naturally occurring flavonoid, which is mainly derived from the rhizomes of *Kaempferol galanga* L. It is widely found in various teas and common fruits [52]. Kaempferol has multiple pharmacological activities, for example, ameliorating renal injury and fibrosis [196], and exhibiting notable effects against cancer [235] and Alzheimer’s disease [52]. Kaempferol promotes melanogenesis and reduces H_2_O_2_-induced oxidative stress in PIG1 normal human skin melanocytes by measuring TYR activity, melanin content, mRNA and protein expression of key enzymes, and expression of related pathway proteins [253]. Kaempferol regulated melanocytes’ dendritic growth and melanosome quantity, maturation, and transport via P38/ERK MAPK and PI3K/AKT signalling pathways [220]. These findings provide a new strategy for promoting hair follicle melanogenesis.

### Gambogic amide

An analogue of gambogic acid, gambogic amide, is the primary active ingredient of the TCM, *Garcinia hanburyi* Hook.f, which was originally identified as a selective tropomyosin receptor kinase A (TrkA) agonist and nerve growth factor-mimetic small molecule [95, 179]. TrkA protein is expressed in the human scalp skin, particularly in the melanocytes and keratinocytes, and is associated with the hair cycle. Strong TrkA expression occurs in the anagen, whereas weak reactivity occurs in catagen and telogen [1]. Interestingly, in cultured human anagen hair follicles, gambogic amide has been shown to prevent gradual pigment loss by increasing melanocyte activation, migration, and dendrite formation, while stimulating hair shaft elongation [25]. These findings suggest the anti-hair greying and hair growth-promoting properties of the selective TrkA agonist, gambogic amide.

### Epimedium brevicornu

Epimedii folium (EF, Yin-Yang-Huo in Chinese) is the dried leaves of *Epimedium brevicornu* Maxim., *Epimedium sagittatum* Maxim., *Epimedium pubescens* Maxim., and *Epimedium koreanum* Nakai [142]. *Epimedium* is widely believed to be a TCM. It has a diverse pharmacology, including ameliorating chronic kidney diseases [280], anti-osteoporosis[33], nerve protection[249], and enhancing periodontal tissue regeneration[275]. EF can also play a role in pigment regulation, although individual components within *Epimedium* species can exert different effects on melanin function. Icariin, as the primary bioactive component of *Epimedium brevicornu* Maxim., has been shown to stimulate melanogenic activity and behave as a strong TYR activator[264]. Conversely, icariside II and icaridin hydrolysate both have a depigmenting effect [288]. Hong et al.[77] showed that EF extract promoted melanogenesis by upregulating the expression of TYR, TRP-1, and DCT through the MAPK/ERK1/2 signalling pathway. Additionally, EF extract increased the number of melanocytes, and promoted the maturation/transferring of melanocytes to pigmentation in co-cultured cells and keratinocytes. These studies show the potential of EF to treat pigmentation disorders and prevent hair greying.

## Conclusion and future directions

The occurrence of hair greying is gradually increasing, which severely affects psychological health and daily life. Despite this, no specific treatment exists for hair greying in modern medicine. Consequently, developing more targeted, effective, and safe therapeutic strategies to reverse hair bleaching is important. In recent decades, notable progress has been made in the use of natural products and their derivatives for treating hair greying. Some innovative natural products and derivatives, such as artemisinin [138], ligustrazine [67], and berberine [69] have become commonly used in clinical practice due to their strong therapeutic effect and stable quality. Natural products and their derivatives are characterised by their high accessibility, low toxic side effects, and diverse biological activities, which have been considered as possible complementary or alternative therapies. In this study, we aimed to deepen the understanding of the mechanisms by which natural products intervene in hair greying. By analysing nearly two decades of relevant research, we identified many natural products and their derivatives that provide new avenues for preventing and reversing unpigmented hair. These compounds can be used alone or in combination with other methods to promote hair recoloration. The therapeutic mechanisms of these natural products have been summarised, including direct regulation of McSCs, melanin production, melanin transport, and related signalling pathways such as the cAMP/PKA and MAPK signalling pathways. Due to the various mechanisms by which natural products and derivatives improve hair greying, they have an expected application prospect, but several perspectives should be noted.

Firstly, most of research on the anti-hair greying effects of natural products and their derivatives are preclinical experiments. However, a large number of natural products and their derivatives are identified that have far-reaching therapeutic promise for different degrees of depigmentated hair. A great deal of work is required to screen and validate the effective compounds.

Secondly, considering the drawbacks such as poor bioavailability, more effective drug delivery ways are required. Therefore, it will take time for them to be rolled out to clinical trials. However, the rapid development of the microneedle and nanomedicine industry is expected to solve this problem in the short term.

Third, the use of natural products and their derivatives may also show toxicity, such as liver and kidney injuries. In recent years, this has been a growing cause of herbal-related adverse effects in America, China, and Japan [17, 183, 213, 284]. In this review, we found that most natural products and their derivatives were mainly administered topically to reverse grey hair. But for these reasons, natural products and their derivatives should be used cautiously in patients with monitoring for signs of toxicity.

Taken together, current research offers novel insights into this prevention and treatment for hair greying, offering key choices for individuals experiencing hair depigmentation. We believe that the development of these natural products and their derivatives into natural medicine, dietary supplements, or haircare products will be an effective way to promote the application process and rapid popularisation.

## Supplementary Information

Supplementary material 1. Table 1. The potential mechanisms by which natural products and their derivatives regulate the proliferation and differentiation of McSCs.

Supplementary material 2. Table 2. The potential mechanisms by which bioactive compounds regulate melanogenesis.

Supplementary material 3. Table 3. The potential mechanisms by which extract of single herb regulate melanogenesis.

Supplementary material 4. Table 4. The potential mechanisms by which pharmaceutical preparation regulate melanogenesis.

Supplementary material 5. Table 5. The potential mechanisms by which natural products and their derivatives regulate melanin transport.

## Data Availability

No data was used for the research described in the article.

## Abbreviations

ADRB2/β2AR: β2-Adrenergic receptor
AKT/PKB: Protein kinase B
cAMP: Cyclic adenosine monophosphate
CREB: CAMP-response element binding protein
DCT: Dopachrome tautomerase
EF: Epimedii Folium
ERK: Extracellular regulated protein kinases
GSK3β: Glycogen synthase kinase-3 beta‌
HMVII: Human melanoma cell line
HEM: Human epidermal melanocyte
MAPK: Mitogen-activated protein kinase
McSCs: Melanocyte stem cells
MITF: Microphthalmia-associated transcription factor
NRF2: Nuclear factor erythroid 2 (E2)-related factor 2
PM: Polygoni multiflori radix
PKA: Protein kinase A
PTU: 1-Phenyl-2-thiourea
TCM: Traditional Chinese medicine
TA: Transit-amplifying
TPA: 12-O-tetradecanoylphorbol-13-acetate
TYR: Tyrosinase
TrKA: Tropomyosin receptor kinase A
TRP-1: Tyrosinase-related protein 1
TRP-2: Tyrosinase-related protein 2
